# Parkinson's Disease in Central Asian and Transcaucasian Countries: A Review of Epidemiology, Genetics, Clinical Characteristics, and Access to Care

**DOI:** 10.1155/2019/2905739

**Published:** 2019-08-08

**Authors:** Rauan Kaiyrzhanov, Mie Rizig, Akbota Aitkulova, Nazira Zharkinbekova, Chingiz Shashkin, Gulnaz Kaishibayeva, Altynay Karimova, Talgat Khaibullin, Dinara Sadykova, Manizha Ganieva, Khurshidakhon Rasulova, Henry Houlden

**Affiliations:** ^1^University College London, Institute of Neurology, Department of Neuromuscular Disorders, Queen Square, WC1N 3BG London, UK; ^2^Al-Farabi Kazakh National University, Department of Biology and Biotechnology, 71 Al-Farabi Ave., 050040 Almaty, Kazakhstan; ^3^South Kazakhstan Medical Academy, Department of Neurology, 1/1 Al-Farabi Avenue, 160019 Shymkent, Kazakhstan; ^4^Kazakh National Medical University, Department of Neurology, 94 Tole Bi Street, 050000 Almaty, Kazakhstan; ^5^Institute of Neurology Named After S.K. Kaishibayev, 9a Mamur 4 Microdistrict, 050000 Almaty, Kazakhstan; ^6^Semey Medical University, Department of Neurology and Neurophysiology, 103 Abai Street, 071400 Semey, Kazakhstan; ^7^Astana Medical University, 49A Beibitshilik Street, 01000 Nur-Sultan, Kazakhstan; ^8^Avicenna Tajik State Medical University, Department of Neurology and Medical Genetics, 139 Rudaki Street, 734001 Dushanbe, Tajikistan; ^9^Tashkent Pediatric Medical Institute, Department of Internal Diseases and Research Laboratory, 223 Bogishamol Street, 100140 Tashkent, Uzbekistan

## Abstract

Our understanding of Parkinson's disease (PD) has significantly accelerated over the last few years, but predominant advances have been made in developed, Western countries. Little is known about PD in the Central Asian (CA) and Transcaucasian (TC) countries. Here, we review the clinical characteristics, treatments used, epidemiology, and genetics of PD in CA and TC countries via a methodological search in MEDLINE, EMBASE, Scopus, Web of Science, and Google Scholar databases. For the acquisition of PD care-related data, the search was extended to the local web resources. Our findings showed that PD prevalence in the region is averaging 62 per 100,000 population. The mean age of onset is 56.4 ± 2.8 in females and 63.3 ± 3.5 in males. Large-scale national studies on PD prevalence from the region are currently lacking. A limited number of genetic studies with small cohorts and inconclusive results were identified. The G2019S LRRK2 mutation, the commonest mutation in PD worldwide, was found in 5.7% of patients with idiopathic PD and 17.6% of familial cases in 153 Uzbek patients. Our review highlighted systematic deficiencies in PD health care in the region including lacks of neurologists specializing in PD, delays in PD diagnosis, absence of specialized PD nurses and PD rehab services, limited access to PD medications and surgery, and the unavailability of PD infusion therapies. Overall, this article demonstrated the paucity of data on this common neurological disorder in CA and TC countries and identified a number of healthcare areas that require an urgent consideration. We conclude that well-designed large-scale epidemiological, genetic, and clinical studies are desperately needed in this region. Healthcare professionals, local and national institutions, and stakeholders must come together to address deficiencies in PD healthcare systems in CA and TC countries.

## 1. Introduction

**Figure 1:**
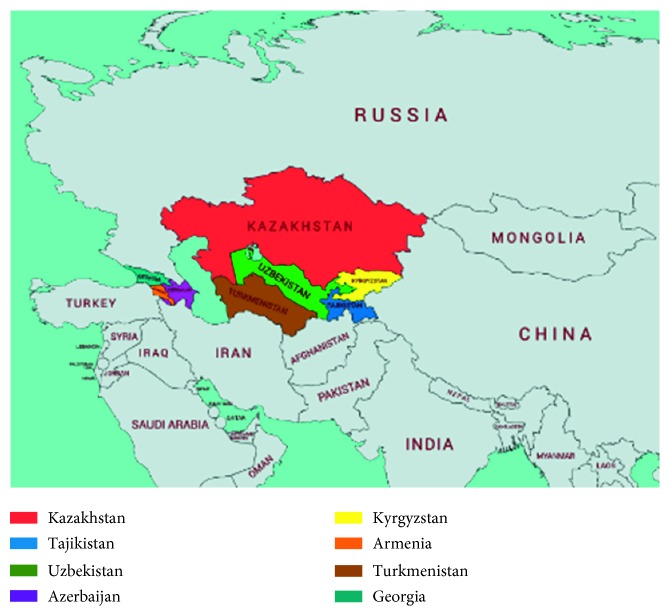
Central Asian and Transcaucasian countries on the world map.

Central Asian (CA) and Transcaucasian (TC) countries consist of a number of independent, upper-middle- and low-middle-income post-Soviet Union countries, populated with diverse ethnic groups. While Central Asia with overall population of 70 million is formed of five countries including Kazakhstan, Uzbekistan, Kyrgyzstan, Turkmenistan, and Tajikistan, Transcaucasian countries include Azerbaijan, Armenia, and Georgia with overall regional population of 16 million (Figure 1, Table 1). The segregation of these countries to Central Asia and Transcaucasia is the result of geopolitical influence after the collapse of Soviet Union [1]. The origin of these ethnic groups, residing in the middle of Eurasian continent, is largely influenced by Indo-Iranian, Turkic, Arabic, and Mongolian expansions and represents a big interest in terms of the genetic diversity of many neurodegenerative disorders including Parkinson's disease (PD). It has recently been reported that the epidemiology and genetics of PD are largely unexplored in these countries [2]. Interestingly, in spite of the moderate proportion of people over 65 years old and the highest median number of neurologists per 100,000 population over the World Health Organization regions [3], very limited PD-related data are available in these countries that show low prevalence of the disease. This might be partially attributable to low public awareness of PD, lack of sufficient knowledge in PD among medical specialists, and inattention to PD-related research. This review pursues the following objectives: providing a summary of all available epidemiologic, clinical, and genetic studies published up to November 2018 from CA and TC countries and reviewing the level and accessibility of PD-related care in CA and TC countries.

## 2. Materials and Methods

### 2.1. Inclusion Criteria

This review encompassed all available population, outpatient clinic, and in-patient-based studies focusing on the clinical, genetic, epidemiologic, and care-related issues of PD in CA and TC regions published up to November 2018. The review included studies focusing only on idiopathic PD using the United Kingdom Parkinson's Disease Society (UKPDS) Brain Bank [4] or Movement Disorders Society (MDS) PD Diagnostic Criteria [5] in ascertaining the PD diagnosis. Studies published only in English and Russian languages were included.

### 2.2. Exclusion Criteria

Publications that did not focus on the frequency, clinical presentation, genetics, and care-related aspects of PD were excluded. Review articles were excluded, with the exception of their references.

### 2.3. Search Methods

MEDLINE, EMBASE, Scopus, Web of Science, and Google Scholar databases were searched for available research articles. “The name of the relevant country within the regions” and “Parkinson's disease” terms were used as keywords for the search, e.g., “Kazakhstan and Parkinson's disease”. With the purpose of including all the pertinent publications, the search was extended to the following resources:

1. Abstracts and poster presentations published in the abstract books of the MDS, International Association of Parkinsonism and Related Disorders (IAPRD), and European Federation of Neurological Societies (EFNS) meetings
2. Reference lists of all the available review articles and primary studies

**Figure 2:**
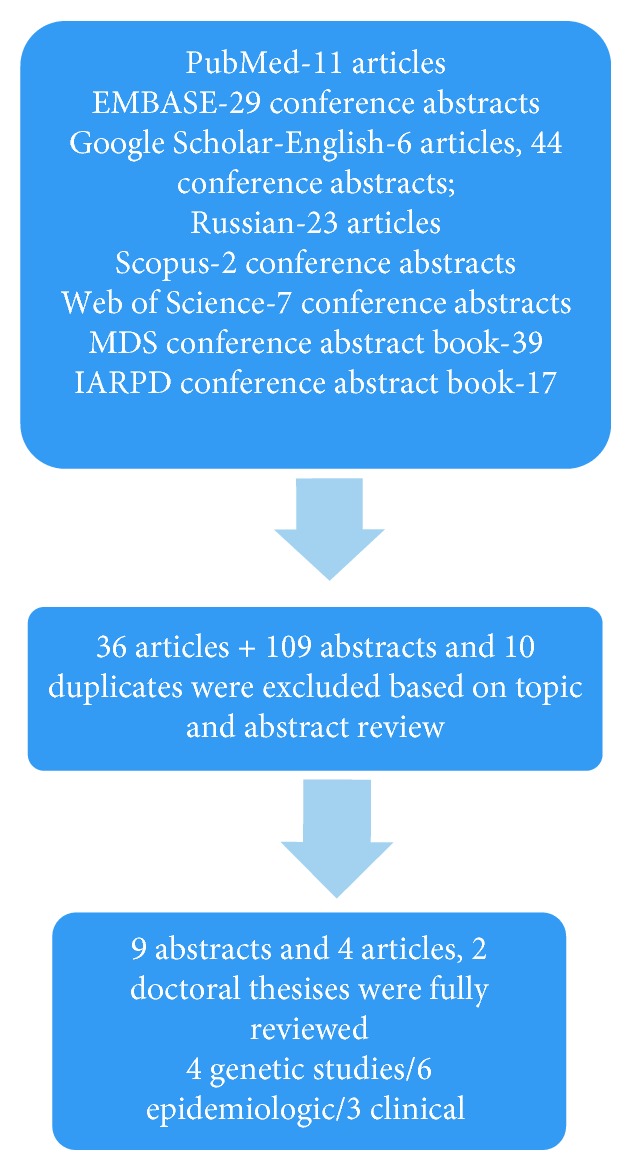
PRISMA flow diagram.

If data reporting the available PD treatment and care in CA and TC countries were not found in the listed databases, the search was extended to the official web resources belonging to the Health Ministries of each country, World Bank (WB) and World Health Organization (WHO) statistics concerning healthcare facilities, MDS website, and any available local reports on PD-related services. PRISMA flow diagram was used to report the data search procedure (Figure 2).

### 2.4. Data Collection and Analysis

The titles and abstracts of obtained publications were reviewed, and studies irrelevant to the aims of this review were excluded. The relevant publications were obtained in full copy and thoroughly reviewed. The following information was systematically sought from the reviewed literature: country, authors, publication year, study design, study year and duration, number of recruited PD patients, basic demographic and clinical characteristics including age at study period, age at onset, disease duration, family history of PD, response to levodopa, MDS UPDRS (Unified Parkinson's Disease Rating Scale) score, and clinical subtype of PD. Age-adjusted and crude prevalence figures were recorded from the prevalence studies. For the genetic studies, the reported genes and mutations, as well as methods for genetic analysis, were extracted. Microsoft Excel 2010 was used for data analysis. The ensuing results were presented in the text and tables.

## 3. Results

### 3.1. Prevalence of PD in CA and TC Countries

No large-scale community-based cross-sectional PD prevalence studies have been identified in CA and TC regions. Only outpatient and inpatient registry-based PD prevalence and frequency data have been hardly elicited from one Kazakhstani doctoral thesis [6] published in Russian language and from several Uzbekistani and Kyrgyzstani abstracts published in MDS Congress abstract books [7–9]. In addition, one research article and one MDS abstract from Uzbekistan have been found to report the analysis of environmental risk factors associated with PD among rural and urban Uzbeks [10, 11]. No PD prevalence data are available from the remaining countries of the concerned regions. Referencing the doctoral thesis of Akanova [6] and her thesis-related conference abstract in English [12, 13] displays some figures concerning the incidence and prevalence of PD in Kazakhstan. Thus, according to her research in 2013, there were 7,628 PD patients registered with the database of the state health statistics, which is based on the International Classification of Diseases, Tenth Revision (ICD-10). Outpatient follow-up registries in 2013 showed the mean PD prevalence of 35.8 per 100,000 population in the whole country. However, in the South and East regions of the country, the prevalence rose to 98 and 99 per 100,000, respectively. This was attributed to the larger population, greater number of neurologists, and greater neurology hospital beds in these two regions in comparison with other regions of Kazakhstan. Regarding the incidence of PD, the author analyses the outpatient follow-up registry data from 2010 to 2014 in two major cities of the country and reports the variable PD incidence rates from 13 to 99 per 100,000 during these 4 years. The author had also conducted a small-scale outpatient registry-based PD prevalence study in 2015, in a sample of Almaty city population, and reported crude prevalence of 62 per 100,000 population for the country. There was less data available on PD prevalence from Uzbekistan and Kyrgyzstan. While one conference abstract reports 278 PD cases revealed from an inpatient database in the period of 2013 to 2014 in Uzbekistan [7], another small-scale outpatient registry-based study in Bukhara region of Uzbekistan reported the prevalence of PD at 24 per 100,000 population in the conference abstract [8]. Similarly, only one abstract report on PD frequency is available from Kyrgyzstan, where 1,098 inpatient subjects were examined during 6 months in 2015 and 78 PD cases were diagnosed [9]. Interestingly, one case-control study in Uzbekistan analyzed environmental risk factors associated with the development of PD in 180 PD patients and 180 healthy controls. In total, 64 factors were analyzed through the designed survey. The results showed that the most significant risk factors for PD were contact with toxins (*P* = 0.0005), well water consumption (*P* = 0.001), and working in the chemical industry (*P* = 0.04) [10]. The study also showed relatively younger PD onset among rural patients. Similar case-control study on 70 PD subjects from Uzbekistan was reported in MDS abstract book. This study showed earlier age of the disease onset among rural PD patients and slight male predominance in comparison with urban subjects [11].

### 3.2. PD Genetic Studies in CA and TC Countries

Four research papers have been found to report genetics of PD in CA and TC regions. The important ones are two doctoral thesis reports published in Russian language in Kazakhstan and Uzbekistan in 2015 and 2017, respectively. In addition, two small genetic reports by other authors also come from these two countries (Table 2).

Akanova [6], in her doctoral thesis from Kazakhstan, reports the absence of Ala30Pro, Ala53Thr, Gly46Lis, and Gly51Asp mutations in the SNCA gene in 34 patients with the onset of PD before the age of 50. Another report from this country investigated one familial case of early onset PD for the presence of GIGYF2 mutations in 7 coding exons and found no positive results [14]. A relatively larger PD cohort was genetically investigated in Uzbekistan by Raimova et al. [15, 17] where 153 PD patients and 80 healthy controls were tested for Ala53Thr mutation in SNCA gene, T240M mutation in Parkin gene, G2019S mutation in LRRK2, C282T polymorphism in Nat2 gene, and polymorphisms in GSTT1/GSTM1 genes. While the study did not find targeted mutations in SNCA and Parkin genes, the G2019S mutation in LRRK2 gene was positive in 5.7% of idiopathic PD and 17.6% of familial PD patients. The C282T polymorphism in Nat2 gene showed significant difference in m/m genotype in PD patients (17.98%) in comparison with controls (6.25%). The significantly increased frequencies of 0/0 genotypes in GSTM1 and GSTT1 genes, as well as their combinations, were noticed in PD patients in contrast to controls. Interestingly, another MDS abstract report from Uzbekistan investigated 67 PD patients from Aral region of the country and found 13 (19.4%) PD patients positive to the SNCA gene mutations and 27 (40.3%) PD cases with autosomal recessive inheritance positive to Parkin mutations [16]. However, the reliability of the latter study remains questionable as no specific mutations and methodology of the genetic analysis have been stated. No population-specific genetic variants have been reported in the regions.

### 3.3. Clinical and Demographic Characteristics of PD in CA and TC Countries

Several studies from Kazakhstan and Uzbekistan reported demographic and clinical details of PD patients in their regions. Three independent studies with relatively large cohorts, up to 595, reported mean age at disease onset 56.4 ± 2.8 in females and 63.3 ± 3.5 in males. The age of the patients at the study periods ranged from 20 to 91 years. All three studies reported female predominance in their cohorts with female-to-male ratio of 1.6 : 1 [6, 7, 18] (Supplementary data (available here)).

### 3.4. The State of Available Care for PD Patients in CA and TC Countries

It was challenging to find a systematic study on the availability of PD care in CA and TC countries. This review explores the state of neurology and neurosurgery workforce, availability of PD medication, and physiotherapy in these countries searching local websites.

### 3.5. Neurological Workforce

CA and TC countries are in the European regions of WHO classification [19]. Therefore, the median amount of neurologists and neurosurgeons per 100,000 populations in CA and TC countries has been estimated to be 6.6 and 15, respectively, of note the highest figure among all the WHO regions [3]. However, one should bear in mind that data on neurological workforce were not available for TC countries and Kyrgyzstan in the WHO atlas. There are no official data on neurologists trained in movement disorders in CA and TC countries; however, local web resources revealed the presence of one neurologist specializing in PD in two TC countries and a couple of movement disorders specialists in Kazakhstan and Uzbekistan [20–23].

No trained PD nurses are available in these regions. One study on 545 patients shows that there is a delay in PD diagnosis after the onset symptoms for 3–3.5 years in Kazakhstan. Moreover, 80% of patients in this cohort were diagnosed with PD only in the advanced stage [6]. This might be due to low public awareness of the disease on the one hand and lack of knowledge on the clinical presentation of PD among medical specialists on the other hand.

### 3.6. Availability of Antiparkinsonian Medication and Deep Brain Stimulation

There have not been any studies systematically investigating the availability of antiparkinsonian medications in the regions. However, the official websites of the health ministries in Uzbekistan, Georgia, and Kazakhstan display the list of antiparkinsonian drugs registered with the State Pharmaceutical Registries [24–26]. In that lists, a number of levodopa plus carbidopa and levodopa plus benserazide only tablet forms, piribedil, pramipexole, and ropinirole among dopamine agonists, rasagiline and selegiline among monoamine oxidase inhibitors (MAO), amantadine, and trihexyphenidyl are available. In the majority of CA and TC countries, only trihexyphenidyl 2 mg tablets and rarely levodopa/carbidopa 250/25 are accessible out of charge, and the remaining antiparkinsonian medications are sold by private pharmacies [27, 28]. Browsing web resources revealed one official clinical protocol for PD treatment in CA and TC countries, published in Kazakhstan in 2016. The protocol is based on the UK NICE guidelines and provides a reasonable guide to local neurologists in the PD treatment. Reference to this guideline showed that apart from standard levodopa plus carbidopa formulations, pramipexole is accessible out of charge in Kazakhstan [27].

While this review did not find any information on the availability of the advanced PD treatments as infusion therapies with Duodopa and apomorphine, there are some reports on the application of deep brain stimulation (DBS) in movement disorders including PD in Kazakhstan from 2013. The conference abstract report by Shashkin et al. [29] states that 117 DBS surgeries were done in PD from 2013 to 2015 in Kazakhstan. To date, there are a couple of centers in Kazakhstan actively practicing DBS [30, 31].

### 3.7. PD Rehabilitation Service

Several web resources suggest that there are some rehabilitation centers specializing on PD physiotherapy in Kazakhstan and Uzbekistan [23, 32]. There is no specialized PD speech and language therapy (SLT) service in the regions.

## 4. Discussion

### 4.1. PD Epidemiology

The outpatient registry-based PD prevalence studies showed low prevalence rates of about 62 per 100,000 population in CA. However, the global burden of PD article by Dorsey, Elbaz, and colleagues from the GBD 2016 Parkinson's Disease Collaborators shows higher figures based on ICD-10 state registry [33]. This discrepancy in prevalence figures might be partially attributable to the limitation of data to the outpatient clinics of only one area of the country in the outpatient PD registry-based studies on the one hand. On the other hand, one should bear in mind that the ICD-10 state PD registries in CA and TC countries might include non-PD parkinsonism cases, due to a lack of PD specialists and potential risk for PD overdiagnosis. Therefore, well-designed population-based multicenter door-to-door studies are warranted to have more reliable data on PD prevalence in these regions. The relatively young mean age of PD onset in these regions, especially in females, warrants case-control studies and identification of environmental and genetic determinants of PD in CA and TC countries.

### 4.2. PD Genetics

This review discovered 4 small-scale studies investigating the genetics of PD in the CA and TC countries. The major finding appears to be a high proportion of LRRK2 G2019S mutation among Uzbeks. This adds new data to Asian PD cohorts, since G2019S mutation has previously been very rarely reported among Asian PD patients [34]. The Uzbek finding concerning G2019S mutation probably requires a replication on a larger cohort, and other CA and TC populations have to be studied on this mutation.

Positive findings on Parkin and SNCA mutations in a small cohort of Uzbek studies suggest that genetic investigations involving larger PD cohorts in CA and TC countries may yield more valuable data on the genetic determinants of PD in these regions. The lack of PD genetic studies in these countries might be attributable to insufficient financial resources, facilities for genetic investigations, and field staff to conduct large-scale studies locally. However, collaboration with world leading genetic institutions with an expertise in PD genetics could potentially provide a good opportunity for CA and TC countries to explore the genetics of PD in their population.

### 4.3. PD-Related Care

The review showed the lack of access to a specialized PD-related care in CA and TC countries. There are very few neurologists specializing in PD with no available PD nurses, SLT, and occupational therapy specialists. There are appear to be two centers practicing PD rehabilitation, one in Kazakhstan and one in Uzbekistan; however, more PD rehabilitations centers need to be established in the regions. Although PD drugs are available for purchase, high prices for dopamine agonists and MAO inhibitors significantly affect access to these medications. DBS in PD has been successfully practiced in the capital of Kazakhstan. However, the large territories of TC and especially of CA countries probably require several DBS centers for adequate access and postsurgical follow-up. The aforementioned discussion points might reflect the quality of PD service in the concerned regions and highlight the important needs and potential actions for the improvement of PD-related care. The latter would be educational activities and training a number of PD specialists. Improving public awareness would also be of importance in the early diagnosis of PD and effective management. There is a need in introduction of PD infusion therapies to improve the management of patients with the advanced PD in the region.

## 5. Conclusions

This review highlights several issues related to PD epidemiology, genetics, and care in CA and TC countries. Data on PD prevalence and genetics are sparse and available only in a couple of CA countries, whereas no data are present in the remaining countries of the regions. The available studies report a relatively young onset of PD suggesting a need for well-designed large-scale epidemiological and genetic studies. Very limited number of neurologists specializing in PD, absence of PD nurses, SLT and rehab specialists, and limited access to PD medication and surgery, devoid of PD infusion therapies, might reflect the inadequate level of PD care in CA and TC countries. Therefore, a major effort from active local medical specialists supported by the world PD experts and movement disorders organizations is warranted to increase public awareness of PD, train field staff, and improve PD-related care in these countries.

## Figures and Tables

**Table 1:** Central Asian and Transcaucasian country profiles (https://knoema.com/atlas/ranks, WHO, WB).

	Nominal GDP 2017, mln	Place World Bank	GPD per capita, USD, 2017	Place WB	Population, 2017	Population aged 65 years (%)	Life expectancy, 2016	Human development index—place, 2017	Health GDP (%), 2015
Kazakhstan	159,407	55	8,838	70	18,037,646	7.0	72.3	0.80–56	3.9
Uzbekistan	48,718	83	1,504	149	32,387,200	4.5	71.3	0.71–103	6.2
Kyrgyzstan	7,565	142	1,220	153	6,201,500	4.5	71.0	0.67–119	8.2
Tajikistan	7,146	144	801	163	8,921,343	3.51	71.1	0.65–125	6.9
Turkmenistan	42,355	86	7,356	79	5,758,075	4.3	67.8	0.71–106	6.3
Azerbaijan	40,748	88	4,132	105	9,862,429	6.0	72.0	0.76–78	6.7
Armenia	11,537	130	3,937	111	2,930,450	11.2	74.6	0.76–81	9.9
Georgia	15,159	116	4,078	108	3,717,100	14.9	73.3	0.78–68	7.9

WHO, World Health Organization; WB, Word Bank; GPD, gross domestic product; USD, United States dollars.

**Table 2:** PD genetic studies in Central Asian countries.

*n*	Genetic mutations studied	Analysis method	Results	Sample size	Country (reference)
1	SNCA gene (Ala30Pro, Ala53Thr, Gly46Lis, Gly51Asp)	PCR, gel electrophoresis	No pathogenic mutations found	34 PD cases	Kazakhstan [6]
2	GIGYF2	PCR, Sanger sequencing	No pathogenic mutations found	Not stated	Kazakhstan [14]
3	SNCA (Ala53Thr), Parkin (T240M), LRRK2 (G2019S), Nat2, GSTT1, and GSTM1	PCR, Sanger sequencing	G2019S was found in 5.7% of idiopathic PD and 17.6% of familial PD patients	153 PD cases, 80 controls	Uzbekistan [15]
4	SNCA, Parkin	Not stated	13 (19.4%) positive to SNCA gene mutation, 27 (40.3%) positive to Parkin mutation	67	Uzbekistan [16]

PCR, polymerase chain reaction; PD, Parkinson's disease.

## Supplementary Materials

Supplementary Materials Appendix A: Parkinson's disease clinical studies conducted in Central Asian and Transcaucasian countries.
