# Supplementary material for: Parkinson's Disease in Central Asian and Transcaucasian Countries: A Review of Epidemiology, Genetics, Clinical Characteristics, and Access to Care
Source: Parkinsons Dis. 2019 Aug 8;2019:2905739. doi: 10.1155/2019/2905739 (PMC6702805; doi:10.1155/2019/2905739)
Supplement: Supplementary Materials — Appendix A: Parkinson's disease clinical studies conducted in Central Asian and Transcaucasian countries. [file 2905739.f1.docx]

**Appendix A.** Parkinson’s disease clinical studies conducted in Central Asian and Transcaucasian countries.

PD – Parkinson’s disease, MCI – mild cognitive impairment, MMSE – Mini-Mental State Examination, MOCA – Montreal Cognitive Scale, MDS – Movement Disorders Society, UPDRS – Unified Parkinson’s disease rating scale, IAPRD – International Association of Parkinsonism and related disorders, RLS – restless legs syndrome, UK BBC – United Kingdom Brain Bank Criteria, FAB - Frontal Assessment Battery, CDT - Clock Drawing Test, HAMD - Hamilton Depression Rating Scale.

|  | **Number of PD patients/**  **controls** | **Investigated PD aspect** | **Results** | **Used scales** | **Country, authors, resource** |
| --- | --- | --- | --- | --- | --- |
| 1 | 43 | Depression and anxiety, cognition | 83.7% depression,  81.4% anxiety  MCI – 75.4% | MMSE, MOCA, Zung Self-Rating  Depression scale, and Spielberger anxiety scale | Kyrgyzstan, Shambetova, MDS abstract |
| 2 | 120 | Severity and progression of PD | Mean onset 56.4 ± 2.8 years in female group and 63.6 ± 3.5 years in male group  Mean age at examination 67 + 4.5 years | UPDRS | Kim and Kamenova, IAPRD abstract |
| 3 | 64 | RLS in patients in and without Dopaminergic therapy | 41 (64%) was on Dopaminergic therapy, 16 (39%) with RLS,  23 without Dopaminergic therapy, 2 (8.7%) with RLS | International RLS Study Group in 2003 RLS criteria, UK BBC | Armenia, Tavadyan, MDS abstract |
| 4 | 120 /120 | Cognition in PD | 10.8% of patients had mild cognitive impairment and 4.7% had severe  dementia. | MMSE, MoCA,  FAB and CDT | Kazakhstan, Manatova, IAPRD abstract |
| 5 | 19 | Dementia | 14 (73.7%) with dementia | MMSE | Uzbekistan, Kalendarev, MDS congress abstract |
| 6 | 27 | Depression, sleep disorders | 96,3% with depression and insomnia | HAMD, complete sleep history | Armenia, Tavadyan, MDS poster presentation |
| 7 | 332 | Non-motor fluctuations | Average age at assessment is 70.8 ±8.4  years (range 38,0-89,3), Age at onset of PD  was 64.2±10.1 years (range 28,0-84,3). | UPDRS | Uzbekistan, Mansurova MDS abstract, |
| 8 | 70 | Age at onset | There is a tendency to an earlier onset of the disease in rural areas compared with urban residents 48.8 ± 9.9 and 53.6±9.9 years respectively. | UPDRS | Uzbekistan Lukmonov |
| 9 | 278 | Age at onset, sex distribution | The predominance of women was revealed (63,4% of which were among females and 36,6% males).  Mean age of the onset is 67 year | UPDRS | Tolobova, 2016, MDS abstract |
| 10 | 595 | Age at onset, severity of PD | Onset in women – 56.4±2.8 and  in men 63.±3.5  Mean age at examination 69.8±7.6 (range 20-91).  2.93% onset before 50 years old.  Female predominance 28.8% vs 16.1% in males.  3.03±3.5 years delay in diagnosis | UPDRS | Akanova, 2015, PhD thesis |
